# Nano metamaterials for ultrasensitive Terahertz biosensing

**DOI:** 10.1038/s41598-017-08508-7

**Published:** 2017-08-15

**Authors:** Dong-Kyu Lee, Ji-Hun Kang, Junghoon Kwon, Jun-Seok Lee, Seok Lee, Deok Ha Woo, Jae Hun Kim, Chang-Seon Song, Q-Han Park, Minah Seo

**Affiliations:** 1 Sensor System Research Center, Korea Institute of Science and Technology (KIST), Seoul, 02792 Republic of Korea; 2 Department of Physics, University of California at Berkeley, Berkeley, California, 94720 USA; 3 College of Veterinary Medicine, Konkuk University, Seoul, 05029 Republic of Korea; 4 Molecular Recognition Research Center, Korea Institute of Science and Technology (KIST), Seoul, 02792 Republic of Korea; 5 Department of Biological Chemistry, University of Science & Technology, 113 Gwahak-ro, Yuseong-gu, Daejeon, 305-333 Republic of Korea; 6 Department of Physics, Korea University, Seoul, 02841 Republic of Korea

## Abstract

As a candidate for a rapid detection of biomaterials, terahertz (THz) spectroscopy system can be considered with some advantage in non-destructive, label-free, and non-contact manner. Because protein-ligand binding energy is in the THz range, especially, most important conformational information in molecular interactions can be captured by THz electromagnetic wave. Based on the THz time-domain spectroscopy system, THz nano-metamaterial sensing chips were prepared for great enhancing of detection sensitivity. A metamaterial sensing chip was designed for increasing of absorption cross section of the target sample, related to the transmitted THz near field enhancement via the composition of metamaterial. The measured THz optical properties were then analyzed in terms of refractive index and absorption coefficient, and compared with simulation results. Also, virus quantification regarding various concentrations of the viruses was performed, showing a clear linearity. The proposed sensitive and selective THz detection method can provide abundant information of detected biomaterials to help deep understanding of fundamental optical characteristics of them, suggesting rapid diagnosis way especially useful for such dangerous and time-sensitive target biomaterials.

## Introduction

Since many intra/intermolecular vibration modes of biomaterials such as protein^1, 2^ and DNA^3–6^ are located within THz spectral range, there has been great interest in research with THz spectroscopic system for biomaterials. Specifically, in contrast to other optical techniques including ultraviolet or X-rays, its non-invasive and non-ionizing properties allow THz technique to be utilized as spectroscopy for even more complex structural biomaterials comprising cells^7^ and tissues^8^ without worrying about thermal fluctuations or other nonlinear side effects. In protein, especially, the conformational information plays a major role in molecular interactions and binding with ligands, which can be analyzed by the THz spectroscopy^9^, since the protein-ligand binding energy is in the THz range as well. However, the detection of such small change of optical property could be limited using THz spectroscopic system as it is, due to its too small absorption cross-section. Recently, metamaterial sensing chip based THz detection techniques have been developed for highly sensitive and selective detections of carbohydrates^10^, chemical compounds^11, 12^, thin sample layer^13, 14^ and microorganisms^15^ and overcoming the limit of sensitivity with typical THz spectroscopic systems. Here, we report THz optical characteristics of three different AI viruses investigated using a highly sensitive THz spectroscopy system assisted by nanoscale metamaterial sensing chips. The sensing chip induces huge THz field enhancement^16^ as much as 50 times, leading to increase the detection sensitivity and selectivity at the same time. Therefore, the three different AI virus samples could be clearly discriminated with respect to their optical parameters. The quantification for one of the virus subtypes was also performed verifying the excellence of the THz nano-metamaterial sensing chip, finally.

**Figure 1:**
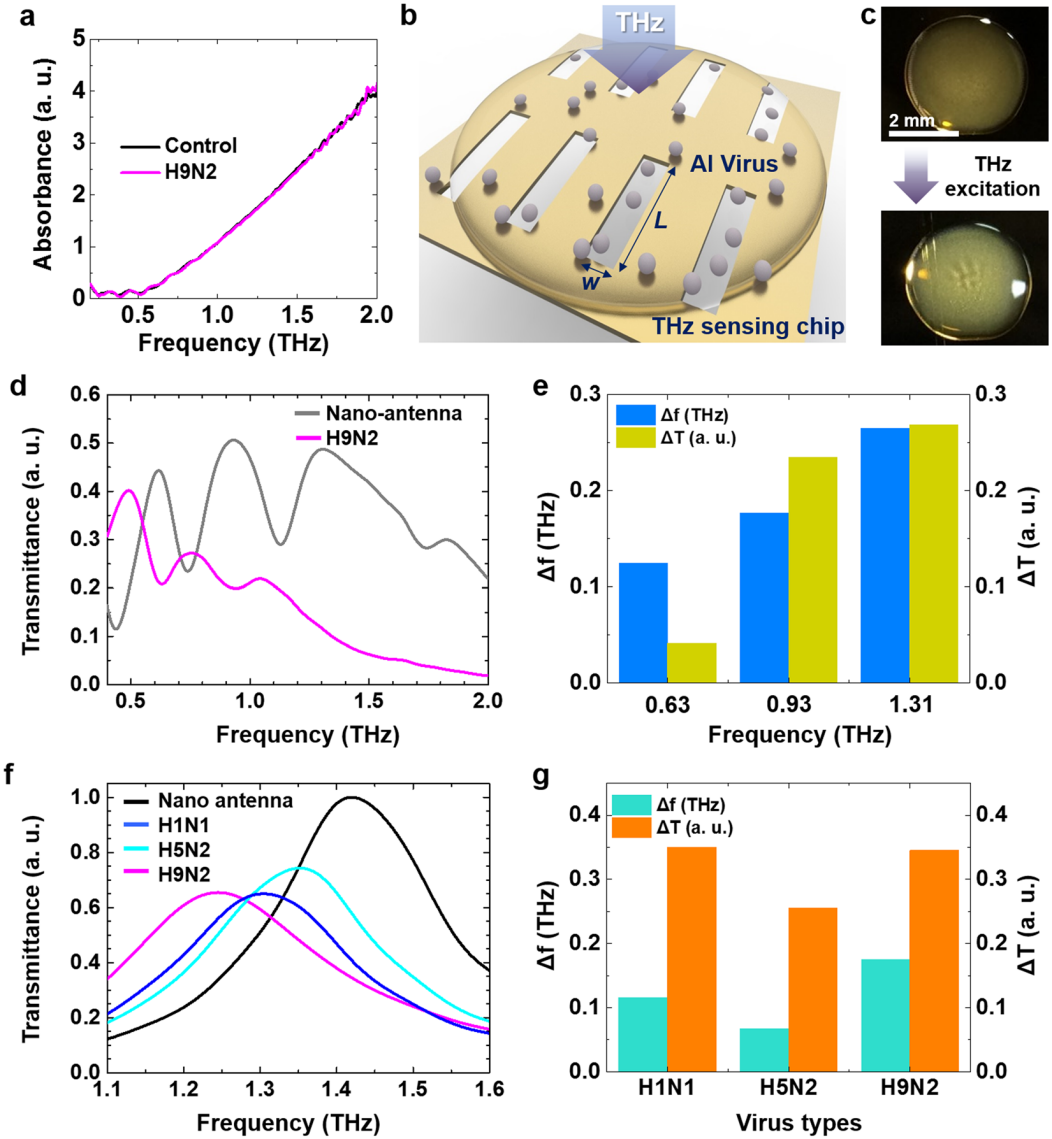
THz detection of virus samples. (**a**) Absorption spectra for pallet types of virus included a protein sample (H9N2) and a control sample without virus in it. (**b**) A conceptual schematic of THz detection of virus samples in liquid state using a nano slot-antenna array based sensing chip. (**c**) Optical images of dropped virus solutions onto the multi-resonance nano-antenna array before (top) and after (down) THz excitation. (**d**) Transmittance spectra through multi-resonance nano-antenna that have three resonance peaks, with and without H9N2 sample onto the antenna. (**e**) The difference in transmitted intensity (*ΔT*) and shifted resonance frequency from each fundamental resonance peak of multi-resonance nano-antenna (*Δf*) for H9N2 sample are represented.

Since several subtypes of Avian Influenza (AI) viruses, frequently causing worldwide outbreaks, are extremely pathogenic, a development of quick and accurate diagnostic methods has been highly demanded (see details in Supplementary Information). In order to investigate fundamental optical characteristics of targeted virus samples, THz transmission measurements without any assistive sensing chip were performed firstly. The targeted three virus samples are: A/NWS/33 (H1N1), A/wild bird/Korea/K09-652/2009 (H5N2), and A/Korean native chicken/Korea/K040110/2010 (H9N2), as shown in Table 1. Among three different virus samples, we chose the H9N2 subtype virus and control (without inoculation of any virus) in pellet forms after freeze-drying process. An absorbance was extracted from the transmission measurement through a pallet type sample in a same way described in ref. 10. The H9N2 and control samples have no recognizable absorption features in the THz spectrum (Fig. 1a) due to superposition of many protein vibration modes and inhomogeneous broadening of absorption features^17^. In contrast to earlier works^10–12^, it is not easy to define fundamental resonance frequency to design nano-antenna based sensing chip for the target bio sample in this case without unique spectral features from their intrinsic modes. In this experiment, therefore, multi-resonance nano-antenna was suggested which is based on the concept of THz nano-antennas with a log-periodic alignment^18^. For an optically unknown target molecules, the multi-resonance nano-antenna is very useful, since it maintains very high sensitivity in ultrabroadband THz regime.

**Table 1:** The strain names with subtype and total protein concentration of each virus samples are represented.

Subtype-strain name	Total protein concentration (mg/ml)
A/NWS/33 (H1N1)	0.54
A/wild bird/Korea/K09-652/2009 (H5N2)	0.2
A/Korean native chicken/Korea/k040110/2010 (H9N2)	0.28

Figure 1b shows a schematic of THz transmittance measurement through the virus sample dropped sensing chip. The multi-resonance nano-antenna sensing chip was fabricated with two-dimensional punctured rectangular slots with a width of *w* = 500 nm, lengths of *l* = 200, 100, 67, and 50 μm, and spacings between adjacent two antennas are 15, 7.5, and 3.75 μm in the horizontal direction, respectively. The second type of nano-antenna sensing chip, used in later measurements, was fabricated with a length of *l* = 40 um. The periods between slots are 40 um in the horizontal direction and 50 um in the vertical direction. These nano-antennas are patterned in a 150-nm-thick gold on the top of double-side-polished 500-μm-thick silicon wafer using an e-beam lithography technique. The total slot numbers more than 1000 within a whole area of 2 mm × 2 mm are designed to minimize possible errors from a random distribution of protein sample during the liquid drop casting. Each slot has a fundamental resonance (*f*_*res*_); for example, the multi-resonance nano-antenna sensing chip has three resonances at 0.62, 0.93, and 1.31 THz, respectively, and the single-resonance nano-antenna sensing chip has a single fundamental resonance at 1.4 THz according to the effective refractive index of substrate^19^. For the measurements of virus samples, we dropped 2 uL of virus solution in liquid state and dried it for 1 hour in controlled laboratory atmosphere (30% of relative humidity and 20 degree of Celsius) to avoid any possible signal error from the water absorption at THz frequency^20^. The dropped virus sample builds a thin film on the multi-resonance sensing chip as shown in the top of Fig. 1c. After 20 minutes of THz field excitation, diffraction pattern appeared in the middle of film as shown in the bottom of Fig. 1c. Without THz field, the pattern disappeared within 5 minutes. Protein aggregation and crystallization effect under high electric field have been reported previously^21–23^. Even though our case is not exactly matched to the conditions for the reported protein crystallization, such as concentration of protein, precipitants, pH, and temperature of protein solution, it can be assumed that the changed surface is related to the temporal aggregation as a beginning step of protein crystallization under strongly enhanced THz electric field. The further study on this interesting effect of protein aggregation and induced crystallization using nano-antenna assisted THz field is needed, but it will not be discussed here in depth (out of scope of this report).

**Figure 2:**
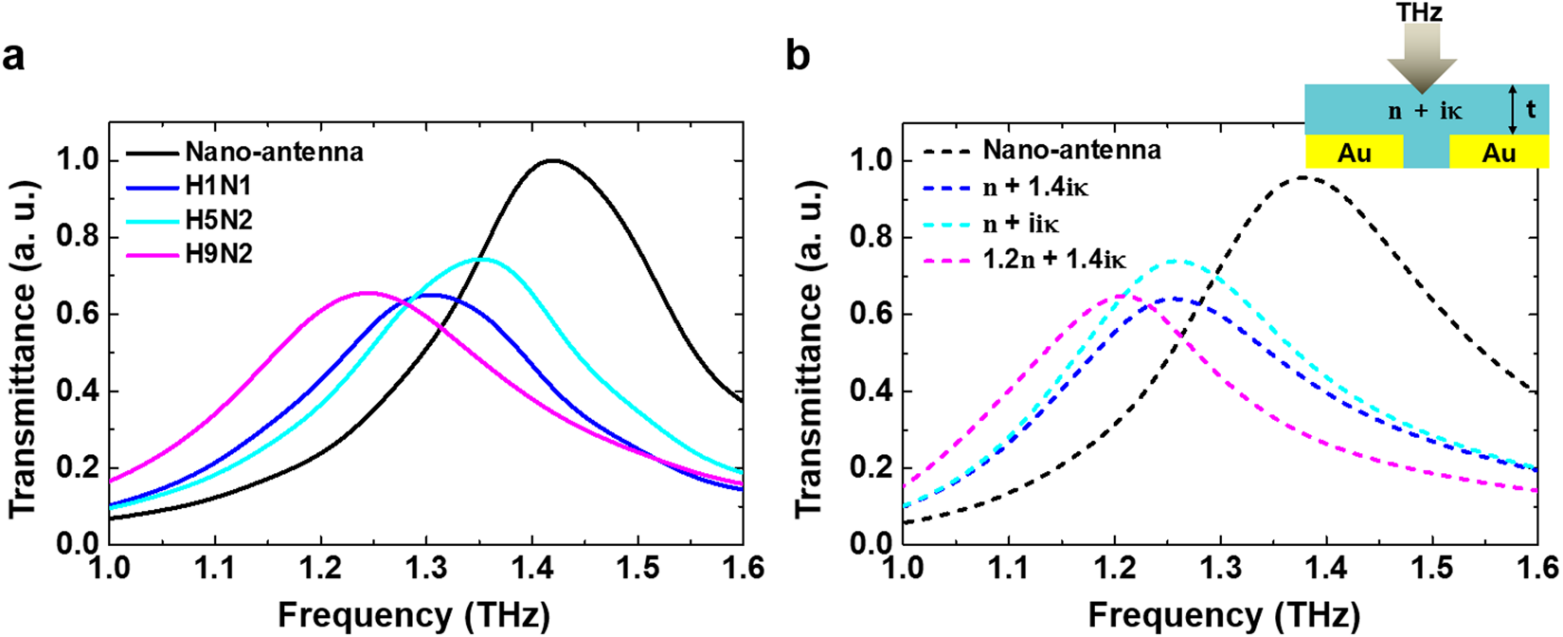
THz optical properties for various types of virus samples. (**a**) Normalized THz transmission spectra measured using the single-resonance nano-antenna without virus sample (black), and with selected three viruses, H1N1 (blue), H5N2 (cyan), and H9N2 (magenta). (**b**) FDTD simulation results of transmittances for the single-resonance nano-antenna and the three different model samples with various composition of dielectric constants (*n* and *κ*) are shown. Inset indicates a used geometry for simulation.

THz optical response for H9N2 virus sample was characterized with transmission measurement through multi-resonance nano-antenna (Fig. 1d). The THz optical responses in terms of the changed values at three maximum transmittances (*ΔT*) and shifted resonance frequencies (*Δf*) are respectively plotted in Fig. 1e. As a meaningful frequency for further measurements, 1.4 THz was selected owing to its biggest changes in both *ΔT* and *Δf* values, implying the highest sensitivity. Further results with 1.4 THz single-resonance nano-antenna in Fig. 2 present that the viruses can be classified in terms of resonance frequency and decreased transmission ratio.

Measured transmission spectra via single-resonance nano-antenna sensing chip with a fundamental resonance frequency at 1.4 THz with and without three AI virus samples are shown in Fig. 2a. Each virus sample shows a distinct transmission change and a shift in the resonance frequency related to their different surfaces and inherent strains. Further analysis and comparison of THz optical characteristics for different subtype viruses were investigated through numerical simulations using the Finite-difference time-domain (FDTD) method (Fig. 2b) (details in Supplementary Information). In FDTD simulations calculating transmission spectra, virus samples are considered as 5 μm-thick, homogeneous dielectric clads possessing different complex refractive indices as illustrated in inset (see the methods). In Fig. 2, experimental results on transmittance characteristics of various samples are compared with numerical FDTD results showing a good agreement. The transmission value change is affected by the molecular absorption accompanying with the change of the imaginary part of the refractive index, *κ* of each sample. Also, the change of the real part of the refractive index, *n* is related to the composition of the virus samples. The spectral changes in transmission, therefore, can be confident evidences to define and identify the virus samples, as well presented in both experiments and simulations.

**Figure 3:**
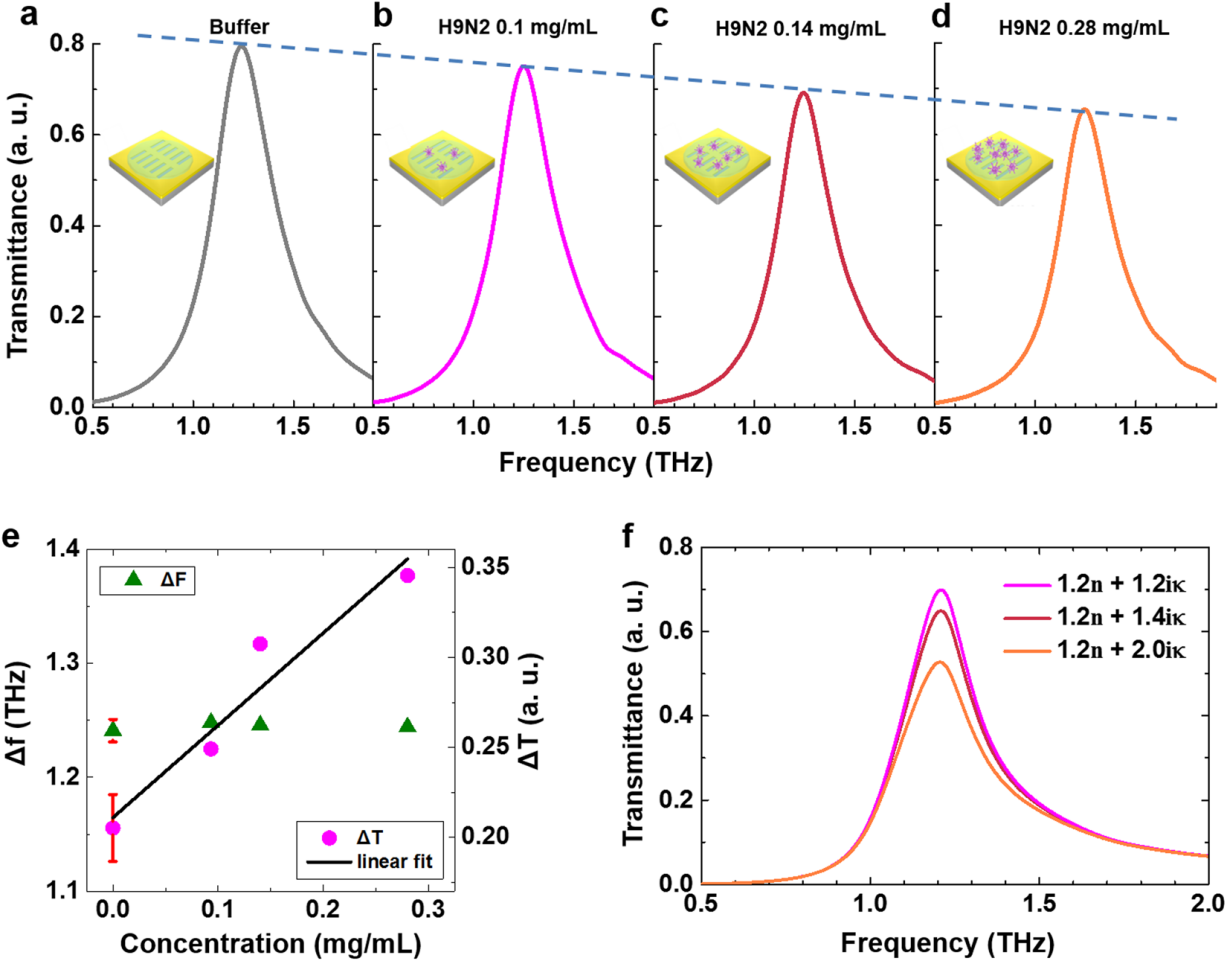
Virus quantification by nano-metamaterial based THz detection. (**a**–**d**) Normalized THz spectra for various concentrations (0, 1, 0.14, 0.28 mg/ml) of H9N2 virus in the buffer solution. (**e**) The changes in the maximum values of the normalized transmittances (*ΔT*, magenta closed circle) and shifted resonance frequency (*Δf*, green closed triangle) are plotted for H9N2 virus in different concentrations as a function of concentration level. The red bar is error bar of buffer solution measurement. Black line and gray dashed line are linear fitting of the transmittance change and frequency shift data, respectively. (**f**) FDTD simulation results of transmittances for three different model samples with various composition of dielectric constants (*n* and *κ*) are shown.

In order to get more insight into detection mechanism using THz sensing chip, concentration dependence was examined for one subtype of the viruses. We measured transmission spectra for H9N2 virus in various concentrations via nano-antenna sensing chip (*f*_*res*_ = 1.4 THz) as shown in Fig. 3a–d. The various concentrations of virus samples were prepared by diluting virus samples (total protein concentration: 0.28 mg/dl) with buffer liquid (total protein concentration: 0 mg/dl) by 1:1 and 2:1 volume ratio, producing four samples: 0, 1, 0.14, 0.28 mg/ml, respectively. The buffer is prepared without inoculation of any virus, as same as the control sample used in pellet experiment. As the concentration of virus increases, the maximum transmittance value decreases, due to its absorption change as shown in Fig. 3e (magenta). It is noted that the clear linearity can be extracted from the data, allowing a promising quantification tool of such protein samples which is one of the critical issues in protein studies. On the one hand, the resonance frequency is maintained, whereas the maximum value of the transmittance was obviously changed in terms of the concentration. The virus-concentration-dependent refractive index was assumed with an effective medium approximation that treats heterogeneous media as homogeneous. The film-likely coated sample on the metamaterial is assumed as 5 μm thick cladding, same as previous FDTD simulations in Fig. 2b, and the densest concentration of protein among three virus samples is 0.54 mg/ml. According to ref. 22, the density of the influenza virus is calculated as 1.104 g/ml^24^, the volume fraction in the coated virus sample on the sensing chip can be estimated as about 0.05%. The used volume fraction is too small to evidently affect the whole refractive index of the sample. Therefore, the resonance frequency is not significantly changed in terms of the concentration increase, besides initial change *Δf* = 0.18 THz was observed from bare antenna to first dropping. The FDTD simulation results (Fig. 3e) also confirm that the transmittance value is mainly changed, whereas the resonance shift is negligible with increasing of *κ*. To exclude the concentration dependency in classifying and subtyping various viruses, considering the normalization per unit mass of virus is essential.

**Figure 4:**
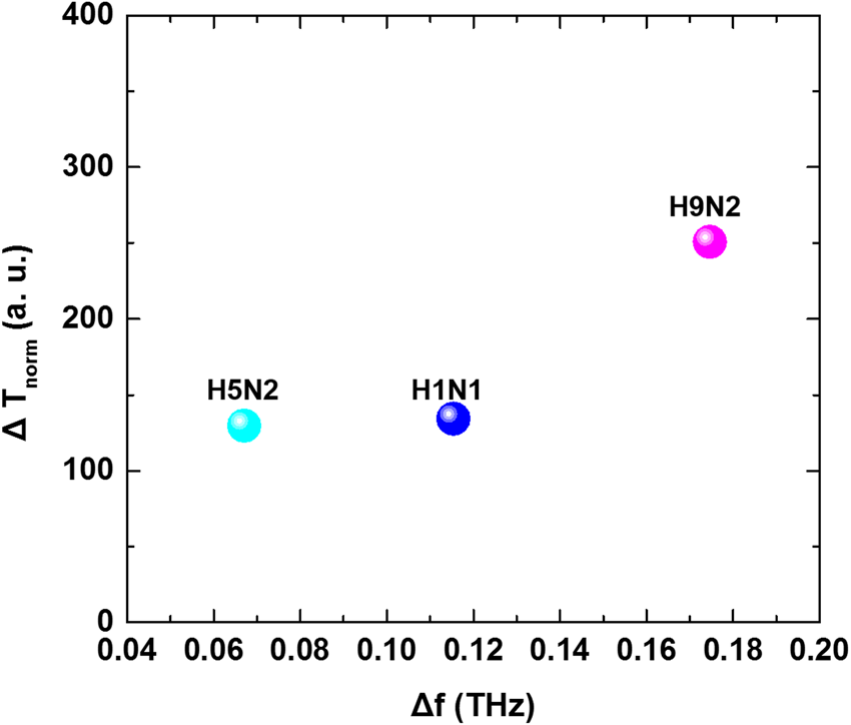
Classified map for various virus samples as functions of the frequency shift and transmittance decrement per unit mass. The three different subtype virus show their own frequency shift and mass-normalized transmittance change.

In order to categorize the subtype of viruses, finally, the THz transmittance spectra via the sensing chips are characterized with two important parameters; the resonance frequency shift and the decrease of normalized transmittance value. The *ΔT*_*norm*_ is again defined with the relationship between the decrease of normalized transmittance value and a mass of virus in the sample as following: *ΔT*_*norm*_ = $\frac{{T}_{buffer}-{T}_{v}}{m}$, where *T*_*buffer*_ is a transmittance maximum value for buffer solution without any protein, *T*_*v*_ is a transmittance maximum value for virus contained protein, and *m* is a mass of virus sample calculated from concentration of virus samples. *Δf* is the shifted frequency from the maximum value of *T*_*buffer*_ to *T*_*v*_. The transmission spectra for three virus samples (H5N2, H1N1, and H9N2) were mapped with two parameters, *ΔT*_*norm*_ and *Δf* (Fig. 4), showing their own frequency shift and mass-normalized transmittance change. For three virus samples, *ΔT*_*norm*_ and *Δf* were extracted from Fig. 2a. Especially, the H9N2 has a same surface protein, neuraminidase, with H5N2, but the location of it in the map is farther than H1N1. This is reasonable with the fact that each same subtype of virus has a same spike shaped protein, located outside of the virus but different strain in it. Therefore, it can be explained that H9N2 is located far from H5N2 in the map, since the difference of strain in the virus may be larger than other subtype of viruses. For the complete mapping of the subtype of the viruses, further substantial experiments with various types of samples to collect vast database will be worth.

In conclusion, The THz optical characteristics of several types of AI viruses were investigated using nano-metamaterial sensing chip with THz spectroscopy. Multi-resonance nano-antenna sensing chip is very useful to detect optically unknown bio samples, especially, as such viruses without their unique fingerprinting at reliable frequency range. This preliminary attempt allows us to select suitable single-resonance nano-antenna optimized for the special virus detecting. The measured THz spectra for various virus samples were analyzed in terms of the optical properties, and discussed with FDTD simulations demonstrating that spectral changes can emerge from the optical properties of samples near the nano-antenna. According to the optical properties including complex refractive index and absorption characteristics, tested viruses could be categorized with respect to their subtypes. Moreover, the virus quantification was successfully performed with a concentration dependence. Introduced nano-metamaterial based THz sensing, here, can provide a quick solution for the detection of AI viruses in non-contact and label-free manner, allowing quantification with very high accuracy additionally.

## Electronic supplementary material

Supplementary for Nano metamaterials for ultrasensitive Terahertz biosensing
