# Supplementary material for: Nano metamaterials for ultrasensitive Terahertz biosensing
Source: Sci Rep. 2017 Aug 15;7:8146. doi: 10.1038/s41598-017-08508-7 (PMC5557762; doi:10.1038/s41598-017-08508-7)
Supplement: Supplementary file 1 — Supplementary for Nano metamaterials for ultrasensitive Terahertz biosensing [file 41598_2017_8508_MOESM1_ESM.pdf]

Supplementary Information for Nano metamaterials for ultrasensitive Terahertz biosensing

Dong-Kyu Lee[1], *Ji-Hun Kang*[2], *Junghoon Kwon*[3], *Jun-Seok Lee*[4, 5], *Seok Lee*[1], *Deok Ha Woo*[1], *Jae Hun Kim*[1], *Chang-Seon Song*[3], *Q-Han Park*[6], *and Minah Seo*[1†]

[1]Sensor System Research Center, Korea Institute of Science and Technology (KIST), Seoul, 02792, Republic of Korea

[2]Department of Physics, University of California at Berkeley, Berkeley, California 94720, USA

[3]College of Veterinary Medicine, Konkuk University, Seoul, 05029, Republic of Korea

[4]Molecular Recognition Research Center, Korea Institute of Science and Technology (KIST), Seoul, 02792, Republic of Korea

[5]Department of Biological Chemistry, University of Science & Technology, 113 Gwahak-ro, Yuseong-gu, Daejeon 305-333, Republic of Korea

[6]Department of Physics, Korea University, Seoul, 02841, Republic of Korea

[†] To whom correspondence should be addressed. [†]mseo@kist.re.kr

1. Influenza A virus sample preparation

Avian Influenza (AI) viruses are divided into subtypes based on two surface glycoproteins, hemagglutinin (HA, H1-H16) and neuraminidase (NA, N1-N9).[25] Some strains of H5 and H7 influenza viruses are then categorized as highly pathogenic avian influenza (HPAI) viruses[26] and they cause high mortality and huge economic losses in the poultry industry. In many countries, as valid control methods, stamping-out strategy for rapid eradication of HPAI viruses[27] and vaccination strategy for control of low pathogenic avian influenza (LPAI) viruses[28] are implemented. Despite such efforts, still a development of rapid and adequate detection tool has an important role to help reduce the handling time for eradication and the economic loss incidental to the outbreak. One of the most popular means to isolate the viruses and subtype them is using embryonated chicken egg that requires an extra time for incubation. Also, molecular detection method such as real-time reverse transcription polymerase chain reaction (rRT-PCR) assays is used for rapid and sensitive detection in broad range of AI viruses.[29] Still it seems that the rRT-PCR has a top priority as a rapid detection technique, but accurate subtyping of the viruses is challenging due to the possible genetic variability of the influenza virus. Moreover, quantification of influenza virus is more difficult with labor-intensive and time-consuming processes.

The AI virus samples (subtype and strain names for used three different viruses are shown in Table 1) were prepared using 10 day old specific pathogen free embryonated chicken eggs at 37°C for 3 days. The allantoic fluids were harvested and chemically treated with formalin (final concentration of 0.2%) for 24 hours at room temperature for virus inactivation. The inactivated allantoic fluids were then centrifuged at 3000Xg for 10 minutes and supernatants were filtered using 0.45 μm syringe filter to remove large cell debris. The solutions were dialyzed with dialysis membrane (Spectra/Por MWCO 1000; Spectrum Laboratories, Houston)

to remove small debris. The total protein concentrations of purified fluid were quantified using Bradford assay (Bio-rad, Hercules, CA).

2. THz time-domain spectroscopy system and transmittance measurement

THz optical properties for prepared viruses were measured using a commercial THz TDS (Time-Domain Spectroscopy) system (Zomega, THz Z-3XL) in the frequency range of 0.5 – 2.5 THz. The system is based on the Ti: sapphire femtosecond laser with 100 fs pulse width and a wavelength centered at 800 nm. A high voltage modulated photo-conductive-antenna (PCA) was used for THz wave generation module and ZnTe nonlinear crystal was used for detection via electro-optical (EO)-sampling method. The THz waves were guided and focused through TPX (polymethypentene) THz lenses and metal mirrors. A pair of TPX THz lenses (10 cm of focal length) focused THz waves with a size of few millimeters and sensing chips with virus samples were mounted at the focal point. The total system was purged with dry air to remove water vapor absorption peak within our measurement spectral range. The measured transmittance, $T = \frac{I_{sam}(\omega)}{I_{ref}(\omega)} = \frac{(E_{sam}(\omega))^2}{(E_{ref}(\omega))^2}$, is obtained from the THz transmittance through a sample attached to an squared aperture with a size of 1.6 mm, $I_{sam}(\omega) = (E_{sam}(\omega))^2$, divided by the THz transmittance through a silicon wafer attached to the same aperture, $I_{ref}(\omega) = (E_{ref}(\omega))^2$.

3. FDTD numerical simulations

We used a home-made FDTD program for the numerical simulations. A non-uniform meshing method with minimum grid-size of 10 nm was adapted to implement deep sub-wavelength thickness of the metamaterial. In our FDTD calculations, virus samples were assumed as non-magnetic lossy clads on the metamaterial, possessing complex refractive

indices $An+iB\kappa$ where A and B are frequency-independent values varied in the range of 1.0-1.2 (for n) and 1.0-2.0 (for κ). n and κ are real and imaginary parts of $\sqrt{\varepsilon}$, respectively, where permittivity ε is modeled as $\varepsilon=1.5^2-\omega_p^2/(\omega^2- \omega_0^2+i\omega\gamma)$ with ω_p=4.0 THz, ω_0=2.8π THz, and γ=4.0 THz. The lossy media were implemented by using auxiliary differential equation method.
